# Accelerating environmental health sciences through data and metadata standards to support FAIR principles

**DOI:** 10.1038/s44573-026-00023-z

**Published:** 2026-11-05

**Authors:** David Hines, Jiang Bian, Patricia Ceger, Nicole C. Deziel, Nomi Harris, Xing He, Hui Hu, Mollie R. Cummins, Sierra Moxon, Christopher J. Mungall, Shuteng Niu, Cui Tao, Sabrina Toro, Alexa Burger, Martin J. O’Connor, Mark A. Musen, Vasilis Vasiliou, Kei-Hoi Cheung, Carol M. Hamilton, Ramkiran Gouripeddi

**Affiliations:** 1 RTI International, PO Box 12194, Durham, NC 27709-2194 USA; 2 Department of Biostatistics & Health Data Science, School of Medicine, Indiana University, Indianapolis, USA; 3 Department of Environmental Health Sciences, Yale School of Public Health, Yale University, New Haven, USA; 4 Environmental Genomics and Systems Biology Division, Lawrence Berkeley National Laboratory, Berkeley, USA; 5 Department of Medicine, Brigham and Women’s Hospital, Harvard Medical School, Boston, USA; 6 College of Nursing, University of Utah, Salt Lake City, USA; 7 Department of Artificial Intelligence and Informatics, Mayo Clinic, Rochester, USA; 8 Department of Genetics, University of North Carolina at Chapel Hill, Chapel Hill, USA; 9 Department of Orthopedics, University of Colorado Anschutz Medical Campus, Aurora, USA; 10 Stanford Medicine, Stanford, USA; 11 Department of Biomedical Informatics and Data Science, Yale School of Medicine, Yale University, New Haven, USA; 12 Department of Biomedical Informatics, University of Utah, Salt Lake City, USA

**Keywords:** Interdisciplinary communication, Metadata standards, Environmental health, FAIR data principles, Information integration, Data sharing, Computational biology and bioinformatics, Ecology, Ecology, Environmental sciences, Health care, Scientific community

## Abstract

Environmental health sciences (EHS) generate varied and highly specialized datasets that span fields including exposure science, toxicology, epidemiology, and molecular biology. Despite their shared relevance to EHS, these data streams can be siloed due to inconsistent metadata practices, limited use of common data elements, and uneven adoption of community standards. This fragmentation limits data integration, reduces reproducibility, and hampers the development and application of emerging computational approaches, including artificial intelligence (AI) and machine learning (ML). This article provides an overview of existing data and metadata standards most relevant to EHS, including public health initiatives, controlled vocabularies and ontologies, and platforms that support findability, accessibility, interoperability, and reusability (FAIR data practices). We outline practical steps for researchers who wish to incorporate standards into their workflows, emphasizing early planning, use of established templates, and availability of institutional resources. We then provide examples of ongoing efforts across EHS domains to develop interoperable infrastructures that enable data integration and highlight how standards enable leveraging of emerging technologies such as AI/ML to enhance data use. This work makes a key contribution to the EHS field by demonstrating the importance of community engagement and active collaboration among developers and users, demystifying the processes of adoption of these tools across EHS domains. As AI/ML technologies stand poised to revolutionize cross-disciplinary science, we provide a vision for the future of an AI-ready, interoperable EHS data ecosystem to support interdisciplinary research, enhance transparency, and accelerate scientific discovery.

## Overview

Environmental Health Sciences (EHS) focuses on how the natural and built environments impact human health and well-being. This field encompasses multiple research domains including toxicology, epidemiology, exposure science, molecular biology and environmental monitoring. Cross-cutting these domains are elements from the social environment, adding to the complexity of EHS challenges. Accordingly, EHS requires an interdisciplinary approach, often generating and integrating diverse data types ranging from environmental contaminant levels and human exposure data, to biological responses at the cellular or molecular level (-omics), gene-environment interactions, to phenotypic observations and human health records^1–3^. These varied data streams offer insights into the complex interactions between environmental exposures and health outcomes but integrating them is often difficult due to disciplinary silos, inconsistent data formats, limited documentation, and challenges in accessibility. As a result of these inconsistencies, EHS data are often not Findable, Accessible, Interoperable, and Reproducible (FAIR)^4^, posing challenges for interdisciplinary analyses that build predictive models and support robust conclusions^5^. For example, experimental approaches seek to understand the modes of action of various types of exposures to develop prophylactics, interventions, and treatments to support human health, but the complexity of integrating these data can hinder EHS investigators seeking to “connect the dots.”

Efforts to harmonize data structures, document methodological details, and provide contextual information are increasingly important for facilitating interoperability and data use in EHS. There is a recognized need for sharing knowledge and data across multiple disciplines to assess when and how people are exposed to hazardous substances^6^, and the National Institute of Health (NIH) 2023 Data Management and Sharing Policy emphasizes the need for consistent, shareable, and comparable data^7^. Furthermore, funding requirements increasingly include the use of data and metadata standards as a necessary component of compliance with requirements. Data and metadata standards help researchers achieve these goals by capturing and organizing information to support integration of datasets from different domains.

Data standards are agreed upon based on rules that define data elements such as variable names, value sets, units of measurement, and reporting formats, often leveraging controlled vocabularies and ontologies to make datasets easier to access, search, and analyze. Metadata standards describe the context of the data including how it was collected, what assumptions were made, and information about limitations and applicability domain that determine how it can be reused. For example, genomic standards like MIAME (Minimum Information About a Microarray Experiment) emerged in response to the need to understand reproducibility in gene expression study results^8^. Minimum information standards are sets of guidelines and formats for reporting data derived by specific high-throughput methods (https://FAIRsharing.org). These standards have become broadly accepted and adopted by journals, funding agencies, and data repositories. This manuscript provides an overview of the current state of data and metadata standards for EHS to demystify the use of these tools. We highlight how data and metadata standards enable the data reuse, reproducibility, and cross-domain data integration that is vital to increasing the impact and translational power of EHS research on human health. This work serves as a practical starting point for researchers—especially those unfamiliar with data stewardship—to engage with developers of standards, comply with funding criteria, and improve scientific understanding through a more collaborative EHS research ecosystem.

## Data and metadata standards in EHS

Prior to the early 2000s, EHS data were heavily siloed, hindering efforts to discover links between environmental hazards and chronic disease and leading to several initiatives to increase data sharing and strengthen cross-domain collaboration in the subsequent decades. For example, the National Environmental Public Health Tracking Program, established in 2002 by CDC to address gaps in environmental health surveillance and integrate exposure and health outcome data, has expanded the capacity to deliver timely, consistent information through shared infrastructure, improved data access, and greater comparability across jurisdictions^9–11^. At the same time, reporting standards such as MIAME^8^ have strengthened data quality in their respective fields. Data and metadata standards have also become central to major public health data collection efforts. The National Health and Examination Survey (NHANES), for instance, has emphasized interoperability and reusability through rigorously standardized data collection protocols and comprehensive metadata infrastructure. Its structured data formats and variable naming conventions have enabled the development of tools like the nhanesA R package, which allows researchers to browse and extract harmonized datasets for secondary analysis and cross-cohort comparison^12^. Furthermore, resources such as FAIRSharing^13^, a web-based portal for sharing curated standards, databases, and policies; BioSchemas^14^, which promotes findability of life sciences data, and Bio2RDF^15^, an open source software to support formatting of bioinformatics data have continued to mature, offering practical support for implementing standards. Controlled vocabularies and ontologies, such as the Gene Ontology (GO)^16^, the Environmental Ontology^17^, the Exposure Ontology^18^, and SNOMED CT^19^, have also continued to grow in use over the past two decades, providing EHS researchers with schemas to eliminate confusion and standardize terminologies across datasets. These vocabularies can be browsed and programmatically queried using ontology portals such as Bioportal^20^ and the Ontology Lookup Service (OLS)^21^. Complementing these vocabularies are data modeling frameworks such as LinkML^22^, which provide a mechanism for binding the values of fields in tables and databases to controlled vocabulary terms.

To leverage these advances, several platforms have been designed to integrate standards into research workflows without requiring extensive technical knowledge, making the use of standards more accessible across EHS. The Biolink Model is an open-source data model, built on LinkML, that can help researchers organize data elements and describe relationships among them in a computable fashion^23^. The Center for Expanded Data Annotation and Retrieval (CEDAR)^24^ provides customizable and machine-readable metadata templates based on community-approved schemas, streamlining the process of capturing high-quality metadata. DataCite^25^, a global non-profit organization, enables researchers to register datasets with persistent Digital Object Identifiers (DOIs) while collecting essential metadata fields to ensure they are findable, citable, and trackable. For data cleaning and transformation, OpenRefine^26^ provides a powerful, open-source solution that is particularly useful for standardizing messy or inconsistent datasets. Collectively, these tools lower the barrier to adopting standards and reduce the manual burden of data integration, which includes annotation, curation, documentation and formatting.

While data and metadata standards are an important part of FAIR data development, they also play a vital role in increasing FAIRness and the trustworthiness of Artificial Intelligence (AI) and Machine Learning (ML), and in particular generative AI (i.e. AI that generates complex content rather than simply predict a value or label). Generative AI encompasses transformer-based deep learning models, including those that operate over omics data (e.g. protein or genome sequences) as well as text. Well-labeled and curated data is typically necessary for the training of generative AI models^27,28^. One of the most well-known AI models in the biosciences is AlphaFold, which relied on meticulously curated data in the RCSB Protein Data Bank gathered over many decades, using well-defined and highly granular data models^29–31^. And more recently, AlphaGenome (a genomic model which predicts tissue-specific regulatory effects of mutations) relies on ontology-labeled sample data collected in large genomics projects such as The Encyclopedia of DNA Elements (ENCODE)^32,33^.

One of the most well known classes of generative models are text-based large language models or LLMs (e.g., GPT-5.3 and Claude Opus-4.6), which are auto-regressive deep learning models that have been pre-trained on massive amounts of existing text representing general knowledge. These pre-trained models can predict and assemble sequences of words that are statistically likely to follow from a given text input. Supervised post-training imbues these models with instruction following capabilities^34^, exhibited in platforms such as ChatGPT, where models provide responses to user-provided prompts.

The fact that the training data for LLMs includes large amounts of generalist and non-science based content might appear to be a disadvantage. However, more powerful models require larger training sets^35^, and models trained exclusively on scientific texts do not perform better on most tasks^36^. Nevertheless, LLMs do not accurately capture all knowledge in the literature, and are prone to a class of errors known as hallucinations, where falsehoods are produced with strong certainty by the LLM^37^. While this was a major problem with early LLMs, newer models exhibit this behavior less frequently, and the behavior is often mitigated through using the LLM as part of a broader agentic system. An agentic system involves a loop, in which the LLM can ‘request’ the use of tools such as web searches, using the result to supplement its own latent knowledge. This kind of hybrid approach between an LLM and a trusted source of knowledge, such as a structured database provides benefits of both the generalist reasoning procedures of AI and the reliability of databases^38–40^. Leveraging these LLM tools responsibly requires use of consideration of their environmental footprint, as both model training and inference can require substantial computational resources and associated energy consumption^41^.

**Fig. 1:**
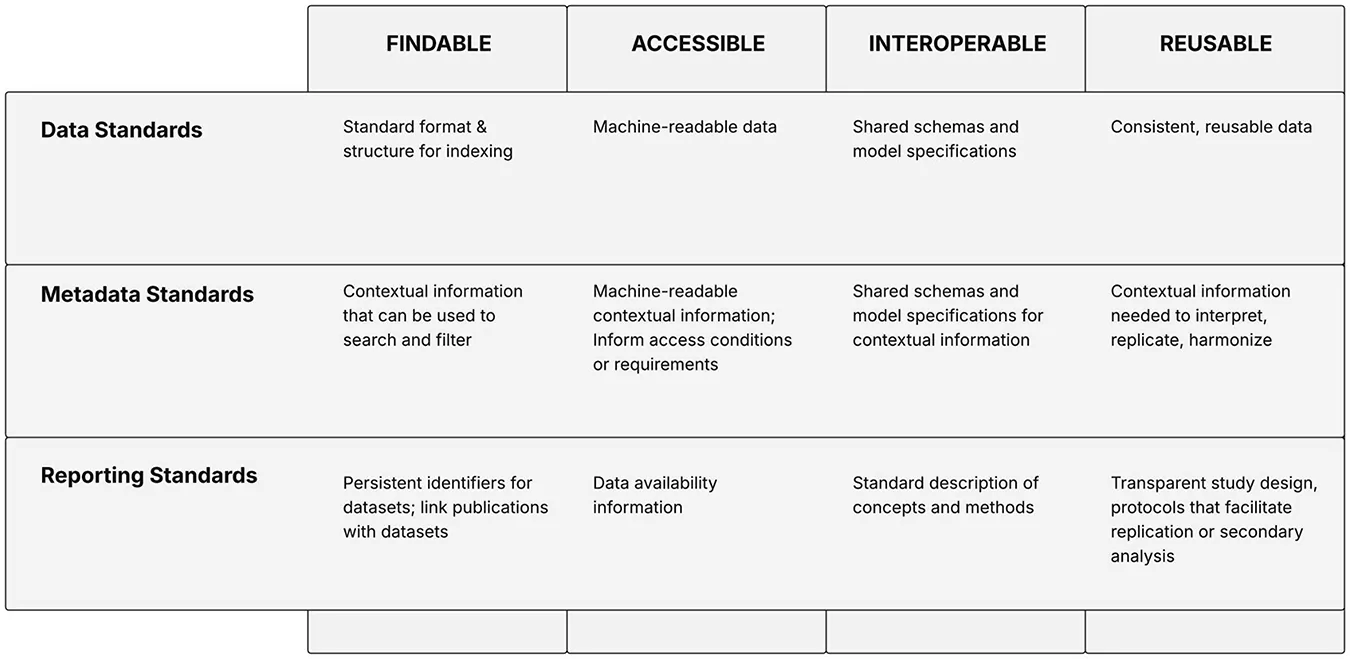
Comparative roles of data, metadata, and reporting standards in enabling FAIR principles.

LLMs and in particular agentic AI provide tremendous opportunities to assist in the construction of ontologies and metadata, with the caveat that results must be carefully checked by experts^42^. Data and metadata standards also include reporting standards and annotation guidelines to help provide the context and structure needed for AI systems to understand, process, and generate accurate, trustworthy, and traceable results (Fig. 1)^43–45^. These principles can be extended to the computational infrastructure that supports AI systems to promote sustainable use of these tools. Energy consumption and the associated emissions are rarely documented in publications using AI and LLMs, which limits our ability to compare and understand environmental impact. Specific AI tools (e.g. small language models) may be better equipped to provide results more sustainably in some research scenarios; thus, as AI-based approaches continue to mature, standardization of environmental impact information such as computational resources and energy use will be important for enabling responsible AI applications^41,46^. Such standards help make data “AI-ready” by making it FAIR, thereby accelerating model development, improving accuracy, and supporting compliance requirements^47^.

## Getting started with data and metadata standards

Data standards are formalized agreements about how information should be structured, formatted, described and exchanged. They provide common data elements (CDEs) which are predefined fields for use across multiple studies to ensure consistency in how specific types of information are collected, described, and interpreted. Successful data and metadata practices begin before any data are collected. Selecting established standards that are relevant to a field, rather than creating ad hoc formats or terminology, is essential for avoiding siloed data. Community-developed standards provide the advantage of incorporating a broad range of use cases and expert knowledge, and thus are particularly effective at enabling researchers in areas of study across EHS to understand and integrate data.

Planning for standards capture early in the research process is also essential to ensuring that critical context, such as relevant methodological details, output variable definitions, and applicability domains can be easily shared and searched^48^. While data standards shape the structure of a dataset, metadata tells the story—what the data are, how they were created, and why they matter. Metadata enables machines to search, identify, and apply experimental results, and enables humans to understand the nuances of data sets to ensure appropriate use and interpretation, thereby enhancing the potential for data integration across EHS research domains. Metadata templates and reporting guidelines are available to help researchers get started and engaged in applying metadata standards to their work. For example, the Organisation for Economic Co-operation and Development (OECD) Harmonised Templates contain structured data elements, picklists for test guidelines, and guidance on the kinds of data required^49^.

Researchers can benefit from connecting with the broader community to improve and maintain data standards. Several international organizations provide leadership, coordination, and ongoing development in this space. FAIRsharing^13^ continues to expand its registry of data standards, repositories, and policies, offering an up-to-date reference point for all disciplines. The International Organization for Standardization (ISO) develops global standards, including many that pertain to data management, quality assurance, and metadata documentation. The World Wide Web Consortium (W3C) maintains web-based standards such as RDF and OWL that enable semantic web as well as linked and interoperable data. The Research Data Alliance (RDA) brings together researchers, funders, and institutions to develop cross-disciplinary data practices and implementation tools. These organizations not only publish guidance and best practices but also offer opportunities for engagement, collaboration, and support as researchers adopt more standardized approaches in their own work.

Here, we provide a broad overview of some of the existing standards that are useful for EHS-related data to help the reader understand which standards might be relevant for their work (Table 1). This overview is not meant to be an exhaustive list of data and metadata standards for EHS related data, but rather a starting place to identify relevant standards readers may want to use. Resources like https://FAIRsharing.org can further assist in identifying the most appropriate standards for a specific discipline.

**Table 1:** A list of select standards and tools that are relevant to EHS data and links to help researchers evaluate whether they are useful for their data.

Resource	Tools type	Primary purpose	Link
OECD Harmonised Templates	Data Standard	Structured templates for reporting chemical properties, toxicology, exposure, and use data for regulatory exchange	https://www.oecd.org/en/topics/sub-issues/assessment-of-chemicals/harmonised-templates.html
ISO 19,115	Data Standard	International standard for geospatial metadata	https://www.fgdc.gov/metadata/iso-suite-of-geospatial-metadata-standards
CF Conventions	Data Standard	Metadata conventions for NetCDF climate and forecast data	http://cfconventions.org/
OECD PBPK Guidance	Data Standard	Regulatory guidance for reporting and evaluating PBPK models	https://www.oecd.org/content/dam/oecd/en/publications/reports/2021/02/guidance-document-on-the-characterisation-validation-and-reporting-of-physiologically-based-kinetic-pbk-models-for-regulatory-purposes_670da2f4/d0de241f-en.pdf
SBML	Data Standard; Data model	Standard for representing computational biological models	http://sbml.org
ISA-Tab	Data Standard	Open source metadata framework for describing investigations, studies, and assays	https://isa-tools.org
Gene Ontology (GO)	Ontology	Controlled vocabulary for gene functions and biological processes	http://geneontology.org
MIAME/ MINSEQE	Data Standard	Minimum reporting standard for microarray/sequencing experiments	https://www.ncbi.nlm.nih.gov/geo/info/MIAME.html
Biolink-Model	Data model; Ontology	Semantic data model for integrating biomedical knowledge graphs	https://biolink.github.io/biolink-model/
CEDAR Workbench	Repository; Data model	Web-based platform for creating machine-actionable metadata templates using ontology terms	https://metadatacenter.org/
RDF / OWL	Ontology; Data Standard	Semantic web standards for ontology and linked data integration	http://www.w3.org/standards/techs/owl
ICD-11	Ontology; Data Standard	International Classification of Diseases diagnostic coding system	https://www.who.int/standards/classifications/classification-of-diseases
OMOP Common Data Model	Data model	Standardized model for observational health data	https://ohdsi.org/data-standardization/
CDISC	Data Standard	Standards for clinical research data submission and exchange	https://www.cdisc.org
STROBE	Data Standard	Reporting guidelines for observational epidemiology studies	https://www.strobe-statement.org
CHEBI	Ontology	Ontology for chemical entities of biological interest	https://www.ebi.ac.uk/chebi/
Mondo	Ontology	Ontology for diseases, harmonizing multiple disease resources	https://www.ebi.ac.uk/ols4/ontologies/mondo
ENVO	Ontology	Ontology that represents environments, environmental processes, ecosystems, etc.	https://www.environmentontology.org/
Exposure ontology (ExO)	Ontology	Ontology for representing exposure data and relationships between exposure events and outcomes	https://www.ebi.ac.uk/ols4/ontologies/exo
Environment Exposure Ontology (ECTO)	Ontology	Ontology for environmental exposures experienced by organisms	https://www.ebi.ac.uk/ols4/ontologies/ecto
Unified Phenotype Ontology (uPheno)/ Human Phenotype Ontology / Zebrafish Phenotype Ontology (ZP)	Ontology	Open source ontologies for representing phenotypes	https://www.ebi.ac.uk/ols4/ontologies/upheno https://www.ebi.ac.uk/ols4/ontologies/hp https://www.ebi.ac.uk/ols4/ontologies/zp
PhenX	Data Standard; Repository	Standardized measures for phenotypes and exposures	https://www.phenxtoolkit.org/
caDSR CDE Repository	Repository; Data Standard	NCI’s repository for Common Data Elements (CDEs)	https://cdebrowser.nci.nih.gov/
MonarchKG	Knowledge Graph	Knowledge graph for cross-species phenotype and disease data	https://monarchinitiative.org/
Exposures KP - icees-kg	Knowledge Graph	Knowledge graph for exposure data	https://stars.renci.org/var/plater/bl-2.1/icees_kgx_files/

## Standards development across EHS

### Infrastructure for source-to-outcome integration

EHS data streams include information from across the source-to-outcome continuum (i.e., the pathway linking environmental sources of exposure to downstream health outcomes), and integrating data from EHS domains requires infrastructure to capture and appropriately address these data types. While approaches such as integrated source-to-outcome analyses can provide powerful insight to support decision making^50^, this type of combinatorial data use requires adherence to data and metadata standards to adequately describe the data and inform the analysis. Development and application of infrastructure to support cross-domain standards is critical for advancing integrative analyses in EHS because they help to support tools that enable broader data use.

For example, a key role of standards is enhancing the ability of knowledge graphs (KGs) (i.e., structured representations of entities and their relationships) to link information from different domains by acting as unifying tools to link information and provide the necessary context. KGs are well suited for organizing biological information because they are highly flexible for representing the complex relationships found in biology, and can be queried to identify relevant measurements, relationships, or data types to facilitate data integration across EHS. Enhancing the ability of researchers to leverage these tools is an area of active research. For example, the Biolink Model^23^, an open-source standard for biological KGs, includes a hierarchy of labeling categories as well as a controlled vocabulary that can function as an upper ontology and contains terms that are common across EHS domains. The Biolink Model evolved to enable the large-scale harmonization of hundreds of biological, chemical, and biomedical data sources, first through the Monarch Initiative^51,52^ and subsequently within the NCATS Biomedical Data Translator ecosystem^53^. It is actively being expanded to encompass additional exposure and dosimetry classes to facilitate integrative analyses ranging from sources of contaminants all the way to health outcomes.

Leveraging frameworks such as the Biolink Model to understand and search relationships among diverse data streams can enable researchers to identify existing data that may be relevant to their work as well as evaluate the appropriateness of those data for integration. As ML and AI driven approaches continue to mature, integrative analyses that make use of multiple data streams are likely to become more common in EHS and related fields. The EHS research community has an opportunity to help promote appropriate applications for data integration through a concerted effort to make contextualizing information machine-readable.

### Case studies

Here, we present case studies of ongoing efforts from The National Institute of Environmental Health Sciences (NIEHS) Data and Metadata Standards program to illustrate how targeted, community-driven solutions can start to close the gaps caused by a lack of shared metadata standards and interoperable data structures across EHS. While each of the projects described operates in a different domain of environmental research, they all face the same fundamental challenge: valuable data are difficult to find, compare, integrate, or reuse. Together, these examples show how the infrastructure provided by data and metadata standards can transform siloed datasets into interoperable resources which support cross-study synthesis, mechanistic insights, and translational research. Each case study highlights a different facet of this goal to build a more connected, transparent, and reusable data ecosystem for EHS.

#### Exposure study reporting template

Synthesizing exposure data is an essential step in environmental health assessments and systematic reviews to address research and policy questions. However, exposure data are often fragmented across repositories and publications, inconsistently structured, and variably documented. In this example, we describe a novel environmental exposure data reporting guideline that was encoded into a machine-readable metadata template to facilitate adherence to FAIR principles.

The reporting template was developed on a structured set of data elements based on a previously published checklist^54^. This set included metadata elements related to study design, study population, exposure assessment method, sampled media. Next, value sets of ontology-based controlled terms were created for individual data elements in this template to support consistent annotation. A workflow that leverages Google Sheets enables domain experts to describe the data elements and value sets in a structured format. These spreadsheets are then converted to (1) a machine-readable metadata template accessible through the Center for Expanded Data Annotation and Retrieval (CEDAR) Workbench, a platform designed to help researchers create, manage, and share structured metadata; and (2) a BioPortal value set. Value sets are designed to bridge gaps in available exposure-related ontologies and primarily built on existing ontology terms, although custom terms were also needed. The resulting exposure study reporting template is publicly available to scientists to submit annotated datasets to appropriate online data repositories including Dryad and Open Science Framework^55^.

#### Spatial and contextual exposome semantic data integration system (SPACESCANS)

Spatial and contextual factors play a critical role in shaping population health, influencing disease risk through interactions with natural, built, and social environments. However, data on spatial and contextual exposures are dispersed across heterogeneous sources, each with distinct formats, spatiotemporal resolutions, and semantic conventions. In this example, we describe ongoing efforts to develop the Spatial and Contextual Exposome Semantic Data Integration System (SPACESCANS) to address these challenges and support downstream applications^56–58^. The system is designed with two core components: (1) a SPAtial and Contextual Exposome Ontology (SPACEO) built from a broad collection of publicly available high-quality spatial and contextual exposure data sets that span natural, built, and social environments; and (2) a linkage tool with a user-friendly interface that allows researchers to connect longitudinal patient data with selected spatial and contextual exposures and appropriate spatiotemporal windows based on study needs.

In practice, the ontology standardizes variable definitions and spatiotemporal scales across data sources, defining semantic meanings for efficient and accurate query. Building upon SPACEO, we are developing a scalable linkage tool with web-based interface that performs spatial matching across different geographic scales (e.g., ZIP Code, census block group), and temporal alignment with longitudinal records from clinical or research cohorts. This infrastructure fills the gap between environmental datasets and real-world clinical and cohort data, enabling large-scale population studies as well as patient-centric analyses. Despite these advances, the heterogeneity of spatial datasets means that not all variables align cleanly with existing ontology terms, and some spatiotemporal mismatches will require manual curation or custom extensions to SPACEO. With SPACESCANS, we successfully linked air quality and water quality exposures with clinical cohorts and developed risk prediction models for gastrointestinal diseases, demonstrating the system’s utility for environmental exposure assessment, disease modeling, and precision public health research^56,59–61^.

#### Zebrafish toxicology phenotype atlas

Zebrafish are a valuable animal model in environmental toxicology due to their ease of breeding, manipulation, and real-time observation of their development^62^. While some labs have their own internal guidelines for standardizing data, these guidelines are usually not shared or used by other labs^63^ and processes for producing and handling data in aquatic studies vary widely. The lack of universally adopted standards is a significant obstacle to using aquatic models in comparative research, including studies on toxicity and health effects (e.g. Adverse Outcome Pathways; AOPs)^64^. Here, we provide an example of efforts to address these challenges through community-driven standards development.

The Zebrafish Toxicology Phenotype Atlas project (ZAPP; https://zappfish.org/*)* aims to develop and promote community-driven standards to improve data compatibility. It leverages a LinkML-based data model for toxicological exposure and their phenotypic outcomes (https://github.com/zappfish/zebrafish-toxicology-atlas-schema*).* Although designed for zebrafish research, this model can be generalizable to other organisms and toxicological assay types. To facilitate and support adoption, an annotation toolkit is under development to allow the research community to annotate their toxicology data according to the data model. Toolkits such as this are essential because they are accessible to users regardless of familiarity with data models or ontologies. ZAPP also aims to actively improve existing ontologies (e.g. Zebrafish Phenotype Ontology and Exposure Ontology) to ensure that the standards used to report phenotypic outcomes and exposure characteristics are available, accurate and specific, and accessible and understandable to the broader research community.

Community engagement is central to the ZAPP project^65^, which is intentionally designed as a community-driven initiative. Because researchers, including but not limited to those in toxicology and EHS domains, are the primary creators and users of toxicology data, their input is essential for defining requirements for the data model and associated ontologies. ZAPP emphasizes a reciprocal model in which researchers articulate needs and constraints and developers translate them into standards and tools that are then refined through continued community feedback. Recent calls for action^66^ illustrate this collaborative approach and invites researchers to help shape the development of ZAPP resources.

#### Sensors and metadata for analytics and research in exposure health (SMARTER)

Current metadata methods for exposure health are essentially ad hoc, leading to data integration and assimilation efforts that are not generally reproducible, a lack of consensus on appropriate metadata standards and conceptual models representing the exposure domain, and investigators and curators of real-world data collections are often not incentivized or responsible for metadata discovery for data re-use. In this example we describe ongoing efforts to address these challenges and provide a consistent approach to metadata capture in exposure health research.

SMARTER^67^ is working to advance community-driven, shared, generalizable metadata and data methods that support reproducible exposure health research, with a special focus on sensor metadata. This project builds upon the Exposure Health Informatics Ecosystem (EHIE)^68^, a comprehensive, standards-based, open-source informatics platform that provides semantically consistent, metadata-driven, event-based management, and simulation and modeling of exposure data. Despite leveraging available standards within EHIE, there are still gaps in sensor‑specific metadata vocabularies and inconsistencies in how sensor characteristics are reported across manufacturers, highlighting the need for more unified and widely adopted community standards.

The SMARTER project leverages a community engagement strategy that includes expert panels, public comment, communications, and user-centered design^69,70^. The project provides an infrastructure for sensor metadata discovery and management^71^, including a graph metadata repository, user-facing tools and artifacts for browsing, selection and submission of sensor metadata, and enabling sensor data use and re-use by rendering the digital objects FAIR. More information about the sensor common metadata specifications, visit: https://github.com/EHIE-CENTER/prisms-sensor-model/.

## Community engagement

To effectively advance data and metadata standards, the community of scientists who generate and use these data must be engaged throughout the process of developing and implementing the standards. By engaging the community and fostering a strong collaboration, the data scientists and engineers who build and manage the data systems are able to align the resulting standards with community goals while researchers gain tools that better support scientific workflows. This engagement of subject matter experts is particularly crucial for research efforts in EHS where studies are diverse and the resulting data are used to inform a range of policy decisions. Community engagement also helps researchers understand how standards and tools can directly support their own work to reduce duplication of effort, improve data quality, and enable more efficient scientific discovery.

The following principles help to ensure the research community members have the understanding and motivation to design studies and contribute high-quality results that address data-related challenges and support future data-driven research projects. In this way, investments in the primary research can be leveraged to facilitate increased impact of the information that is generated. Based on our own experiences, these principles can guide how the community can be supported while adopting new standards and tools, ensuring that researchers are not simply asked to comply with new practices, but are equipped to understand their utility and benefit.

- Community-centered goals: The process should prioritize the community’s vision and goals, using data and metadata to support such goals.
- Transparency and communication: It should foster open and genuine conversations/discussions with the public about data and metadata projects, making project information freely and easily accessible for input and feedback.
- Representation: It should ensure that the community is representative and provides training to empower everyone to understand and contribute to data and metadata initiatives.
- Benefit: The engagement should provide a clear benefit to everyone involved, leading to a culture where public involvement is embedded in the process.
- Two-way interaction: It should encourage active participation where the community is not just a source of information but an active partner in decision-making and implementation, and where developers incorporate community feedback into iterative improvements. This reciprocal model ensures that standards are grounded in real scientific workflows, while providing tools that will reduce the burden of data management and increase the long-term value and reusability of their work.

Adhering to the above principles in community engagement can provide several advantages. Community-driven data and metadata standards promote improved data quality and relevance because these tools and formats are targeted towards the community’s specific needs. Additionally, when a research community is involved in standards development, there is enhanced trust and ownership of the resulting outputs, increasing the likelihood that the standards will be adopted and increasing the data literacy of the community to help them understand, use, and leverage data and metadata for their own purposes. By aligning data and metadata efforts with community goals, assessments can be better designed, monitored, and evaluated for effectiveness and the resulting tools, systems, and projects are more likely to be sustainable and meet long-term objectives, highlighting how critical community involvement is for data and metadata standards development.

Ultimately, guiding the community towards shared data and metadata standards and tools is not about imposing new requirements, but about enabling a more efficient, connected, and impactful research ecosystem, in which researchers, developers, and data users all benefit from the added structure, interoperability, and integration. Below we provide examples of current and ongoing engagement with various EHS communities to develop and implement effective data and metadata standards and highlight the benefit these practices can have for cross-cutting research.

### The national institute of environmental health sciences superfund research program

The NIEHS Superfund Research Program (SRP)^72^ supports research at universities and small businesses to: (i) solve complex environmental health problems, (ii) reduce hazardous contaminant cleanup costs, (iii) decrease exposure to contaminants, and (iv) improve human health. It also provides evidence-based practical, scientific solutions to protect health, the environment, and communities. NIEHS has funded 23 multi-project research centers through SRP P42 grants across the country.

Beyond its role as a funding mechanism, the NIEHS Superfund Research Program represents a uniquely integrative research ecosystem that spans exposure science, toxicology, epidemiology, remediation science, and community engagement. This structure makes the SRP an ideal testbed for implementing FAIR-aligned data and metadata standards across the full source-to-outcome continuum. Because Superfund research is explicitly problem-driven and solution-oriented, standardized and interoperable data are essential not only for scientific reproducibility, but also for regulatory translation, site prioritization, and community-informed decision-making. The overarching goals of the Superfund Research Program are to advance interdisciplinary EHS, support problem-based and solution-oriented research, and develop tools that mitigate the impacts of hazardous substances on human health and ecosystems. Achieving these goals increasingly depends on the integration of robust data and metadata standards into research workflows, enabling harmonization across projects, comparability across sites, and reuse of data beyond individual studies or funding cycles.

As part of the SRP community, the Yale Superfund Research Center (YSRC)^73^ is dedicated to identifying health risk factors associated with emerging drinking water contaminants and finding solutions that help affected communities resolve contamination issues. A key threat to the environmental health of drinking water supplies is toxic, persistent, and water-soluble chemicals. The YSRC focuses on the study of 1–4 Dioxane (and its co-occurrents) and has integrated projects investigating exposure, health risk, environmental monitoring and mitigation, as well as dynamic administrative and research cores that foster research translation and community engagement^74^. Importantly, the YSRC illustrates how integrated data and metadata standards can function as a unifying layer across heterogeneous Superfund projects^72^. By harmonizing exposure measurements, analytical chemistry data, biological response data, and community engagement outputs^75–77^ under shared metadata frameworks, Superfund Centers (SCs) can generate datasets that are reusable beyond individual contaminants, sites, or study designs. This approach enables cross-center comparisons, supports cumulative and mixture-based risk assessment, and creates data resources that are amenable to advanced analytics and AI-driven discovery.

The YSRC community engagement strategy is to engage the SCs to enhance data standards and tools development, while addressing the emergent water contaminants initiatives. The SCs serve as hubs for collaboration among researchers, community stakeholders, and policymakers in the EHS community. To strengthen the two-way nature of engagement, SC researchers provide domain expertise and real-world use cases, while developers translate this input into practical standards that are refined on an iterative basis based on community feedback. Cross-center initiatives can be launched to pool resources and expertise for the training, development, testing, and validation of comprehensive metadata/data standards and tools. Because SCs generate longitudinal, multimodal data spanning environmental measurements, biological responses, and social and community contexts, they are particularly well positioned to benefit from AI- and machine learning–enabled analyses. However, such approaches depend on consistent metadata, transparent provenance, and well-defined exposure and outcome variables. Embedding FAIR-aligned standards within Superfund data workflows ensures that these rich datasets are not only reusable, but also suitable for advanced applications such as exposure pattern discovery, predictive toxicology, and site-specific risk modeling.

Taken together, the SRPs offer a scalable, community-engaged model for embedding data and metadata standards into environmental health research. By aligning FAIR principles with problem-driven science, Superfund provides a pathway for accelerating translation from exposure characterization to regulatory action and public health impact.

### US environment protection agency per- and polyfluoroalkyl substances

US EPA has a long history of funding and conducting observational exposure studies to measure environmental agents that people may contact during their daily activities. These studies are conducted to understand potential impacts of exposure to environmental contaminants on human health. Currently, EPA is committed to addressing Per- and Polyfluoroalkyl substances (PFAS) in drinking water. PFAS are a class of manufactured chemicals of concern due to their widespread production and use, as well as their mobility and persistence in the environment^78^. Most people in the United States have been exposed to some PFAS^79^, with high exposures measured in people impacted by a concentrated source over long periods of time (e.g. Agency for Toxic Substances and Disease Registry,^80^).

To support this work and enable Agency decisions and actions, US EPA develops and aggregates a variety of environmental and health data, including information on PFAS occurrence.

Much of the research about PFAS exposure pathways to date has focused on drinking water, with less known about the role of other pathways, such as residential exposure through house dust and indoor air^81,82^. To more thoroughly characterize the scope of PFAS exposure and pathways, scientists have examined increasingly more diverse media (e.g. Chang et al. 2025 and DeLuca et al. 2024,^83,84^), conducted larger sampling campaigns^84,85^, and applied expanded analytical techniques such as nontargeted analysis (e.g. Boettger et al. 2025,^86^).

Currently, the ability to glean insights across multiple studies is limited by the lack of both a common database to house environmental exposure information and standardization of data characteristics^18,54,87^. To address this need, tools are also being developed to collate information on PFAS in the environment (e.g., *PFAS Analytic Tools*^88^; *Data - NC PFAS Testing Network*^89^. Missing from these resources are observational exposure data for PFAS including measurements in residential media such as tap water and house dust. As such EPA initiated development of a repository to house harmonized, deidentified exposure data to enable cross-study analyses. The resulting tool, Observational PFAS Access paneL (OPAL) includes critical exposure science data elements identified by scientists who are conducting PFAS studies, and is being used to collate available PFAS residential exposure data^90^.

As part of standard metadata template development, efforts are underway within the NIEHS Data and Metadata Standards program to pilot the incorporation of PFAS lists published by EPA and OECD. Efforts have also begun to use LLMs to automate extraction of metadata related to PFAS exposure studies from scientific literature and advance visualization of the extracted results. These tools support a two-way engagement model where researchers provide domain expertise and validation, while developers refine automated methods to better reflect study designs and metadata needs.

EPA is actively addressing PFAS through a range of regulatory authorities (*Key EPA Actions to Address PFAS*^91^. Recently, EPA designated two PFAS, PFOA and PFOS, as hazardous substances under the Comprehensive Environmental Response, Compensation, and Liability Act (CERCLA), also known as Superfund (*Designation of Perfluorooctanoic Acid (PFOA) and Perfluorooctanesulfonic Acid (PFOS) as CERCLA Hazardous Substances*^92^. CERCLA is designed to prioritize sites that present risk to human health and the environment. Designation enables EPA (and other agencies with delegated CERCLA authority including NIEHS) to address prioritized sites. The SRP provides research to inform EPA decisions and assist communities near hazardous sites. Engagement with the SRP research community and EPA regulatory scientists will be important for further advancing metadata/data standards and tools development to enable insights on PFAS environmental health. In addition to SRP and EPA, it will also be important to engage with other communities including those with data repositories (e.g., All of Us^93^ and Bridge2AI^94^ to facilitate the alignment of our environmental data and metadata standards effort with these communities.

## Vision for the future

Many researchers, especially those new to the data and metadata standards, find the landscape of these tools to be burdensome and overly technical. While the importance of good data practices is recognized^95,96^, they are not always implemented, in part because their benefit is unclear, terminology is unfamiliar, tools are fragmented, and guidance may be inconsistent—especially for those in smaller EHS fields without established norms. However, as data and metadata standards continue to mature and tools continue to develop and become more accessible and user friendly, we envision a scenario where cross disciplinary data sharing becomes an integral part of impactful EHS research, and where AI helps researchers navigate, annotate, and organize data with far less manual effort.

Scientific research is a long-term investment – yet without proper metadata and adherence to data standards, even the most carefully generated data can become unusable over time. Formats become obsolete, file structures are forgotten, and undocumented variables lose their meaning. Metadata provides the context needed to preserve datasets so that they remain interpretable and valuable years and even decades after a project concludes. Meanwhile, standards ensure that data can be integrated into future tools, repositories or analyses without requiring reformatting or translation. Well-documented data also becomes more discoverable – both by search engines and by researchers seeking data to support new hypotheses, models or policy decisions. By applying standards and metadata practices up front, researchers increase the scientific impact of individual studies, protect their investment in data, prepare data for use by AI-systems which depend on structured, high-quality inputs to generate reliable insights, and ensure their work’s value for future technologies and collaborators.

Data and metadata standards are increasingly tied to compliance with funding and publishing requirements. They ensure that EHS data are meaningful, reusable, and durable by providing the structural and contextual consistency needed to support reproducibility and reusability. While navigating the landscape of standards can appear daunting and technical, a growing number of tools, community-developed templates, and practical examples are available to help identify relevant standards and make implementation manageable. Embracing these standards early in the research planning process streamlines compliance and helps researchers to promote the long-term value and credibility of scientific work. Additionally, adopting data standards enhances collaboration, visibility, and scientific impact by making it easier to share data across disciplines, integrate findings into larger datasets or consortia, and increase the likelihood of citation and reuse. Embracing data and metadata standards is an approachable step that investigators can take to make a strategic investment in a more connected and reusable future for scientific research, and to prepare their data for an evolving landscape in which AI tools are becoming increasingly common. As researchers adopt AI systems to assist with data processing, exploration, integration, and hypothesis generation, the need for harmonized, well-annotated, AI-ready data will grow. High quality standards ensure that EHS datasets can be used reliably in workflows, supporting trustworthy model development, cross-domain integration, and automated scientific discovery.

## Data Availability

No datasets were generated or analysed during the current study.
